# A Versatile Strategy for Surface Functionalization of Hydrophobic Nanoparticle by Boronic Acid Modified Polymerizable Diacetylene Derivatives

**DOI:** 10.3389/fchem.2019.00734

**Published:** 2019-11-01

**Authors:** Shiwei Gu, Chang Guo, Hui Wang, Guangjun Tian, Suying Xu, Leyu Wang

**Affiliations:** State Key Laboratory of Chemical Resource Engineering, Beijing University of Chemical Technology, Beijing, China

**Keywords:** boronic acid, photo polymerization, surface functionalization, cancer cell targeting, fluorescence imaging

## Abstract

The flourishing advancements in nanotechnology significantly boost their application in biomedical fields. Whereas, inorganic nanomaterials are normally prepared and capped with hydrophobic ligands, which require essential surface modification to increase their biocompatibility and endow extra functions. Phenylboronic acid derivatives have long been known for its capacity for selective recognition of saccharides. Herein, we demonstrated a versatile surface modification strategy to directly convert hydrophobic inorganic nanocrystals into water-dispersible and targeting nanocomposites by employing boronic acid modified photo-polymerizable 10,12-pentacosadiynoicacid and further explore its potentials in selective cancer cell imaging.

## Introduction

In the past two decades, the vast advancements in nanotechnology significantly boost their application in biomedical fields, by taking advantage of rich choices of inorganic nanoparticles that possess as outstanding optical, magnetic, and electrical features. For instance, luminescence nanocomposites have been utilized for molecular recognition, real-time tracking and cell labeling (Liu et al., 2012; Chen et al., 2015; Lu et al., 2015; Arshad et al., 2016; Guo et al., 2019; Nifontova et al., 2019). Magnetic nanomaterials have been employed for magnetic resonance imaging, magnetic guided drug delivery (Mahmoudi and Hadjipanayis, 2014; Zhang et al., 2018; Zhu et al., 2018). Photothermal responsive nanocrystals have displayed great potentials in light-guided drug delivery and photothermal treatment of tumors (Lu et al., 2017; Zhao et al., 2017; Wang et al., 2018; Vines et al., 2019). Whereas, these inorganic nanomaterials are normally prepared in organic solvents and capped with hydrophobic ligands, which require essential surface modification prior to further use. To this end, various methods (Chen et al., 2008; Nurunnabi et al., 2010; Dong et al., 2011; Lu et al., 2011; Deng et al., 2012; Huang et al., 2013) have been developed such as ligand exchange, layer-by-layer strategy, silanization, amphiphilic polymer coating, biomimetic functionalization, *in-situ* polymerization, and so on. Ideally, surface engineering shall well-maintain intrinsic properties of inorganic nanocrystals as well as convert them into hydrophilic, biocompatible and functional nanocomposites, meanwhile, the surface modification protocols shall be simple, straightforward without tedious procedures. In this regard, it still lacks versatile and time-efficient surface engineering strategy that allow for directly converting hydrophobic inorganic nanocrystals into water-dispersible and functional nanocomposites for biomedical utilization.

**Figure 1:**
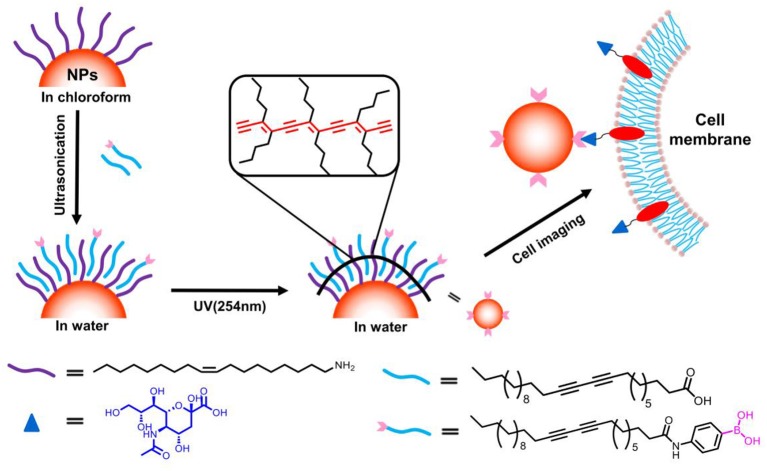
Schematic illustration for surface coating of hydrophobic nanocrystals with boronic acid modified PCDA and its potential for selective cancer cell imaging.

With respect to biomedical application, the specific targeting ability was of crucial importance. Apart from enhanced permeability and retention effect of nanomaterials, the active targeting is always required for site-specific labeling and drug delivery (Choi et al., 2011; Zhao et al., 2015; Hui et al., 2019; Li et al., 2019; Sun et al., 2019; Yan et al., 2019). Phenylboronic acid derivatives have long been known for its capacity for selective recognition of saccharides through covalent formation of boronate ester with 1,2 and 1,3 *cis*-diols presenting on many saccharides (Xu et al., 2010; Nishiyabu et al., 2011; Wang et al., 2014; Sun et al., 2018). As such, fluorescent boronic acid derivatives were developed to monitor glucose variation and detect glycated proteins. Moreover, recent findings revealed that changes of glycan expression on cell surface are closely related with tumor metastasis. For instance, sialic acid moieties have been found to over-expressed in many cancerous cells (Matsumoto et al., 2010). On basis of such observation, boronic acid moieties have been utilized as site-specific labels for targeting cancerous cells (Liu et al., 2011; Wu et al., 2015). Inspired by these achievements, we proposed to use photo-polymerizable 10,12-pentacosadiynoic acid derivatives containing boronic acid moiety (PCDA-BA) for hydrophobic inorganic nanocrystals surface engineering, which in one aspect, would directly convert hydrophobic nanocrystals into water-dispersible ones and in another aspect, endow the resulting nanocomposites with site-specific binding properties (Figure 1). The results demonstrated that the photo-polymerizable property of PCDA allows for straightforward phase transfer of various types of inorganic nanoparticles, suggesting the generality of this method, and moreover, as a proof-of-concept, such surface modification strategy could be readily tailoring the surface functions with boronic acid moieties, resulting in selective labeling of cancerous cells.

## Materials and Methods

### Materials

10,12-Pentacosadiynoicacid (PCDA), *N*-Hydroxysuccinimide (NHS) and sodium stearate were purchased from Alfa Aesar. Carbodiimide (EDC), hydrofluoric acid (HF), 1-dodecanethiol, and indium (III) nitrate trihydrate was obtained from Aladdin. Oleic acid (OA), oleylamine (OAm), and 1-octadecene (ODE) were purchased from Alfa Aesar. 4-Aminophenyl boronic acid hydrochloride was gained from Sukailu Chemical Reagent Company. 2,2-Dimethyl-1,3-propanediol and silver acetate were purchased from Sinoreagent Chemical Reagent Company. Sulfur and Na_2_S·9H_2_O were gained from XLHG Chemicals Co. Fe(NH_4_)_2_(SO_4_)_2_·6H_2_O and NaOH were purchased from Beijing Chem Works. ZnCl_2_·4H_2_O, Cu(NO_3_)_2_·5H_2_O, and AgNO_3_ were obtained from Tianjin GuangFu Chemicals Co., Tianjin FuChen Chemicals Co., and Innochem Chemicals Co., respectively. *N,N*-Dibutyldithiocarbamic acid (HS_2_CNBut_2_) is obtained from Beijing keao Technology Co., Ltd. All the reagents were of analytical grade and used as received without further purification.

### Characterization

TEM images were obtained on a Hitachi H-800 transmission electron microscope operating at 200 kV. Powder XRD patterns were carried out on a Bruker AXS D8-Advanced X-ray diffractometer with Cu Kα radiation (λ = 1.5418 Å). Absorption spectra were obtained using a UV-3600 UV–*vis*–NIR spectrophotometer (Shimadzu) equipped with a plotter unit. Optical properties of the NPs were characterized using a Hitachi F-4600 fluorescence spectrophotometer equipped with a plotter unit and a quartz cell (1 cm × 1 cm). DLS measurements were carried out using a Zetasizer Nano-ZS90 zeta and size analyzer from Malvern. All ^1^H and ^13^C NMR spectra were recorded on a Bruker Avance 400 MHz spectrometer.

### Synthesis of Ag Nanoparticle (Ag NPs)

Six milliliters of ODE and 4 mL of OAm were added into a 50 mL three flask and heated up to 190°C under nitrogen atmosphere. Then the sliver precursor was quickly injected and maintained at this temperature for 30 min. Then solution was cooled to room temperature naturally, and the product was collected by centrifugation. The final products were redispersed in 2 mL of chloroform. The sliver precursor obtained by dissolving silver acetate in ethanol.

### Synthesis of Au Nanoparticle (Au NPs)

Similar to that for preparation of Au NPs, 6 mL of ODE and 4 mL of OAm were added into a 50 mL three flask and heated up to 160°C under nitrogen atmosphere, followed by quick injection of gold precursor, which maintained at 160°C for 30 min. The solution was cooled to room temperature, and the product was collected by centrifugation. The final products were redispersed in 2 mL of chloroform. The Au precursor obtained by HAuCl_4_ dissolved in ethanol.

### Synthesis of Cu_7_S_4_ Nanoparticle

Six milliliters of ODE and 4 mL of OAm were added into a 50 mL three flask and heated up to 190°C under a protective nitrogen atmosphere. Then the precursor of copper was injected into the three flask and maintained this temperature for 15 min, which was cooled to room temperature, and the products were collected by centrifugation. The final products were redispersed in 2 mL of chloroform. The Cu precursor obtained by Cu(NO_3_)_3_ and (HS_2_CNBut_2_) dissolved in ethanol.

### Synthesis of NaYF_4_:Yb^3+^-Er^3+^ Nanoparticle

Firstly, prepared of NaYF_4_:Yb^3+^-Er^3+^ nanoparticles, sodium stearate (0.35 g), a blended solution of OA (7 mL), and ODE (7 mL) were added into a three-necked flask under ultrasonic condition. Then, the HF–oleylamine solution (1.05 mL) and the precursor of rare-earth solution (3 mL) were fastly injected into the flask and stay at 90°C for 20 min. Subsequently, the reaction solution was rise temperature to 180°C and hold 10 min. Finally, the reaction solution was heated to 310°C and kept for 1 h under a protective nitrogen flow. The solution was cooled down to 80°C and through centrifugation the products was obtained. The final products were redispersed in 2 mL of chloroform.

### Synthesis of Fe_3_O_4_ Nanoparticle

Fe_3_O_4_ nanoparticles were prepared by the hydrothermal method. NaOH (1 g) and ultrapure water (6 mL) and ethyl alcohol (10 mL) were added into a 50 mL beaker. Subsequently, OA (10 mL) were injected dropwise. Meanwhile, Fe(NH_4_)_2_(SO_4_)_2_·H_2_O (0.784 g) dissolved into of H_2_O (10 mL) was dropwise added into the beaker, and the transparent solution rapidly became turbid. Finally, the suspension was transferred into a teflon lined autoclave (45 mL) and hydrothermally treated at 220°C for 6 h. After naturally cooling to room temperature, the thus-formed black precipitates were separated through centrifugation, and the final products were redispersed in 2 mL chloroform.

### Synthesis of ZnS-AgInS_2_ (ZAIS) Fluorescent Quantum Dot

ZnS-AgInS_2_ quaternary fluorescent quantum dots were prepared *via* hot-inject method. Firstly, the 0.1025 mmol of indium (III) precursor, 0.5 mmol of ZnCl_2_, 10 mL of ODE, and 1 mL of DDT were added into a 50 mL three flask, then the mixture was heated up to 140°C and kept at this temperature for 15 min under an N_2_ atmosphere. Secondly, 2 mL of sulfur precursor with various concentrations was quickly injected into this solution and heated up to 200°C. After 10 min, 2 mL of silver precursor was injected into the three flask, then the mixed solution was elevated to and maintained at 180°C for 60 min. Finally, the products were centrifuged at 3,000 rpm for 6 min to remove large particles. Fluorescent ZnS-AgInS_2_ quantum dots were separated from the supernatant by methanol, followed by centrifugation for 10 min at 8,000 rpm. The final products were redispersed in 2 mL of chloroform.

Indium (III) precursor was obtained by mixing 1 mmol of indium (III) nitrate trihydrate with 12 mmol of stearic acid at 140°C for 4 h, then naturally cooled down to room temperature. The silver precursor was prepared by dissolving 0.25 mmol of silver nitrate in a mixture solvent of 9 mL of EtOH of and 1 mL of OAm. The sulfur precursor was acquired by dissolving sublimed sulfur (1.6, 2.4, 4.8, and 9.6 mg) in mixture solution containing 1 mL of OAm and 1 mL of ODE.

### Synthesis of 4-Aminophenylboronic Acid Modified Diacetylene Monomer (PCDA-BA)

The synthetic scheme for PCDA-BA (Compound **4**) was listed in Supplementary Figure 1. Specifically, 300 mg (2.61 mmol) of NHS and 750 mg (2 mmol) of PCDA were dissolved with 20 mL of CH_2_Cl_2_ in 50 mL three flask and stirred for 30 min, thereafter, 800 mg (4.17 mmol) of EDC was added under nitrogen flow and stirred at room temperature for 16 h. Then the mixed solution was filtered, followed by evaporation of CH_2_Cl_2_, and the product was redispersed in 25 mL of CHCl_3_ and washed it four times with saturated NaCl aqueous. After that the solution was evaporated, the compound **1** was obtained.

Secondly, 347 mg (2 mmol) of 4-aminophenylboronic acid HCl and 500 mg (4.71 mmol) of sodium carbonate were dissolved in 25 mL CHCl_3_ and stirred at room temperature for 30 min, thereafter, 208 mg (2 mmol) of 2,2-dimethyl-1,3-propanediol and 1,000 mg (7 mmol) sodium sulfate anhydrous added into this solution under a protective nitrogen flow stirred at room temperature for 3 h. After filtering the insoluble inorganic salts, compound **2** (423.7 mg, 95%) was obtained via evaporation under reduced pressure. ^1^H NMR (400 MHz, CDCl_3_) δ(ppm) 1.01 (s, 6H), 3.76 (s, 4H), 6.70 (d, *J* = 8.4 Hz, 2H), 7.65 (d, *J* = 8.3 Hz, 2H).

Finally, the compound **1** and **2** were dispersed in 25 mL of CH_2_Cl_2_ and stirred at temperature for 24 h. After that the solvent being evaporated, compound **3** (642.3 mg, 64%), was obtained by silica gel column (CH_2_Cl_2_/CH_3_OH, 90:5). ^1^H NMR (400 MHz, CDCl_3_) δ(ppm) 0.86 (t, *J* = 6.9 Hz, 3H), 1.03 (s, 6H), 1.24–1.55 (m, 32H), 2.21–2.27 (m, 4H), 2.38–2.45 (m, 2H), 3.7 (s, 4H), 7.51 (d, *J* = 8.3 Hz, 2H), 7.79 (d, *J* = 8.2 Hz, 2H).

Compound **4** (PCDA-BA) was obtained by further treating compound 3 (561 mg, 1 mmol) with aqueous potassium hydrogen fluoride (3 mL, 5.64 mmol) in MeOH (7 mL) under stirring at 25°C for 0.2 h, which was concentrated by rotary evaporator and recrystallized by hot acetone (minimal volume to dissolve solid) and ether to afford corresponding potassium trifluoroborate solid. The potassium trifluoroborate solid (0.5 mmol, 277.5 mg) and sodium carbonate (1.5 mmol, 158.9 mg) were dissolved in water (5 mL) and acetonitrile (10 mL). The mixture then was stirred for 20 h under 25°C. Then a mixed solution of saturated aqueous NH_4_Cl (8 mL) and 1 M HCl (2 mL) was added. The resulting solution was extracted by ethyl acetate (3 × 10 mL). All of organic extracts were dried over sodium sulfate, filtered and concentrated under vacuo to afford PCDA-BA. ^1^H NMR (400 MHz, CD_3_OD) δ(ppm) 0.86 (t, *J* = 6.7 Hz, 3H), 1.23–1.57 (m, 32H), 2.18–2.26 (m, 4H), 2.34–2.41 (m, 2H), 7.56–7.72(4H); MS (ESI, negative ion); calc. C_31_H_48_BNO_3_,m/z 493.37, found, 492.36.

### Procedures for Preparing Hydrophilic Nanocomposites Containing Single NPs

In a typical experiment, 0.005 mol of PCDA and 5 mg hydrophobic nanoparticles (Ag NPs, Au NPs, Fe_3_O_4_ NPs and ZAIS) dissolved in 1 mL of chloroform, was added in a 10 mL of 0.004 M NaOH aqueous solution under ultrasonication (350 W, 12 min). Then, the chloroform was evaporated at 50°C for 2 h. Finally, hydrophilic nanoparticles were washed by ultrapure water and collected by centrifugation at 10,000 rpm for 10 min, which was redispersed into water, followed by UV irradiation treatment under 254-nm UV lamp for 30 s. With respect to preparation of ZAIS@PCDA-BA, all the experiment conditions are identical only that 0.005 mol of a mixture of PCDA and PCDA-BA (PCDA-BA:/PCDA = 1:8) was used instead of pure PCDA.

### Preparations of Nanocomposites Containing Multiple Nanoparticles

Hydrophilic nanocomposites containing multiple NPs were prepared with protocols similar to that containing single nanoparticle, but with variation of NaOH amount being used. Briefly, 0.005 mol of PCDA and 5 mg hydrophobic nanoparticles dissolved in 1 mL of chloroform then the solution was added in 10 mL of 0.001 M NaOH aqueous solution under ultrasonication (350 W, 12 min). Secondly, the chloroform was evaporated at 50°C for 2 h. Finally, hydrophilic nanocomposites were washed by ultrapure water and collected by centrifugation at 10,000 rpm for 10 min and redispersed into water and under 254 nm UV lamp irradiated 30 s.

### Cytotoxicity Experiment

Briefly, HepG2 cells attached to the bottom of 96-well-cell culture plate were incubated with different concentrations (0–300 μg/mL) of sterilized ZAIS@PCDA-BA NCs in each well at 37°C for 24 or 48 h, respectively. Then the cytotoxicity was measured by the methyl thiazolyltetrazolium (MTT) assay (Guo et al., 2018).

### Cell Imaging

HepG2 cells were seeded in a glass bottom cell culture dish and cultured at 37°C overnight. Then the ZAIS@PCDA-BA NCs stock solution was added with a final concentration of 300 μg/mL. The HepG2 cells were incubated with ZAIS@PCDA-BA NCs for different times (2, 4, and 8 h). As a control, the ZAIS@PCDA NCs were incubated with the HepG2 cells for 8 h. Meanwhile, to verify the targeting ability of boronic acid moiety, 0.1 M D-fructose was added as competitive component and co-incubated with were incubated with the HepG2 cells for 8 h in the presence of ZAIS@PCDA-BA NCs. Then, the cells in glass bottom cell culture dish were washed with PBS (pH 7.4, 10 mM) and mixed with 4% paraformaldehyde solution for 15 min. The luminescence imaging was conducted on TCS-SP8 confocal laser microscopes (Leica) equipped with a 488 nm laser.

## Results and Discussion

### Surface Modification of Hydrophobic NPs

**Figure 2:**
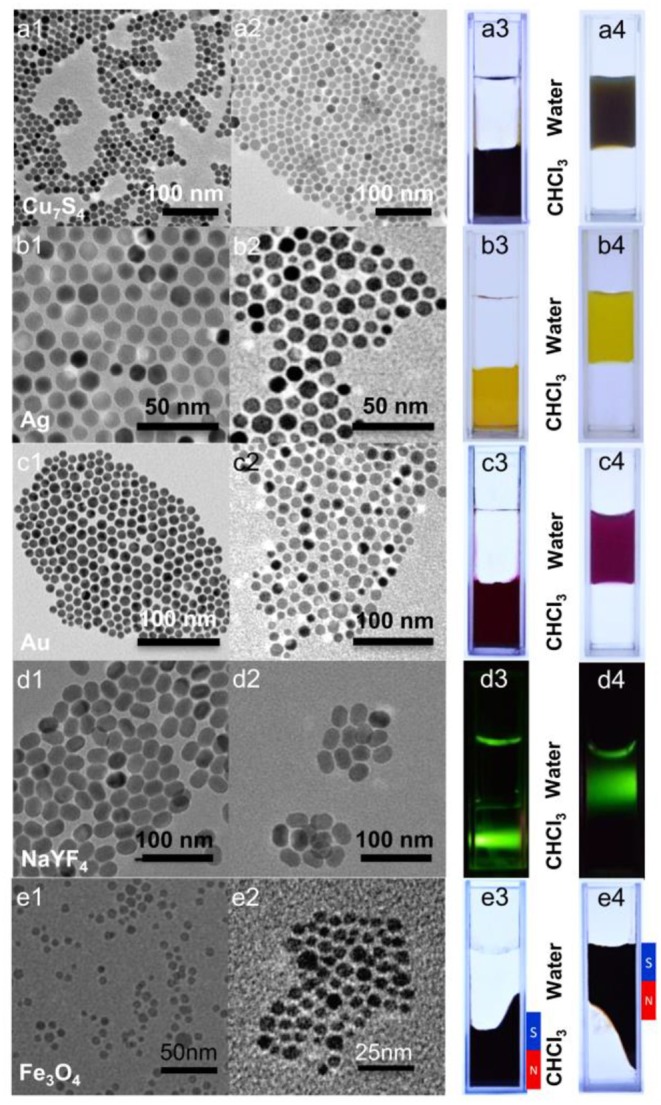
TEM images **(a1–e1, a2–e2)** and photographs **(a3–e3, a4–e4)** of various types of inorganic nanocrystals **(a)**: Cu_7_S_4_, **(b)**: Ag NPs, **(c)**: Au NPs, **(d)**: NaYF_4_:Yb^3+^/Er^3+^, and **(e)** Fe_3_O_4_ before (1, 3) and after (2, 4) surface engineering. Photograph 3 and 4: the top layer is water and the bottom layer is chloroform; **(a–c)** daylight; **(d)** 980-nm diode laser; **(e)** daylight with magnet.

In view of the biomedical application of inorganic nanocrystals, a prerequisite is proper surface modification, which ensures biocompatibility as well as functionalities. 10,12-pentacosadiynoic acid (PCDA) has long been used to form polymerized liposomes under UV irradiation and meanwhile, the polydiacetylene moiety can be a signaling unit, responsive toward external stimuli such as pH (Song et al., 2001), surfactants (Chen et al., 2010; Lee et al., 2011), and metal ions (Lee et al., 2009; Wen et al., 2016). Given that hydrophobic nanocrystals normally were capped with ligands contains long alkyl chain, we assumed 10, 12-pentacosadiynoic acid (PCDA) may serve as a phase transfer agent through hydrophobic-hydrophobic interaction. Indeed, such assumption was demonstrated to transfer hydrophobic upconversion nanoparticles into aqueous phase (Nsubuga et al., 2018). Moreover, the photo-polymerizable properties of PCDA could further anchor surface ligands as well as providing active sites for functionalization. Bearing this mind, initially we testify the feasibility of PCDA for phase transfer. In this regards, as shown in Figure 2, we adopted a series of hydrophobic nanoparticles, including noble mental (Au, ~15 nm; Ag, ~12 nm), inorganic mental sulfides (Cu_7_S_4_, ~12 nm), upconversion nanoparticles (NaYF_4_: Yb^3+^-Er^3+^, ~25 nm), and magnetic nanoparticles (Fe_3_O_4_, ~4 nm) to transfer them into aqueous phase, respectively. Hydrophobic nanoparticles and pristine PCDA were dissolved in chloroform and further added into NaOH aqueous solution to form emulsion under ultrasonication treatment. Under this progress, the PCDA spontaneous assembled onto the surface of hydrophobic nanoparticles. To achieve stable hydrophilic nanocomposites, the resulting solution was then irradiated under 254 nm UV-lamp, which can make PCDA anchored on the surface of hydrophobic nanoparticles through *in-situ* formation of polyacetylene. The strategy for hydrophobic nanoparticles surface engineering is facile and versatile. As evidenced by the results in Figure 2, transmission electron microscope (TEM) images indicated that the size and shape were maintained after surface engineering without aggregation being observed. Particle sizes measured by TEM were smaller than confirmed by dynamic light scattering (DLS), which likely caused by the carboxylic groups of PCDA interacted with water molecular and generated hydration layers. All types of hydrophobic nanocrystals were successfully transfer into aqueous phase without observable interference on the functional properties of inorganic nanoparticles including luminescence properties (Figure 2d) and magnetic properties (Figure 2e). Notably, all of hydrophobic nanoparticles were encapsulated in a one particle per micelle manner since the DLS sizes of nanocrystals merely changed before and after phase transfer process, shown in Supplementary Figure 2. It is worth noting that encapsulation of multiple nanoparticles could be readily achieved through variation the dosage of NaOH being used, as suggested in Supplementary Figure 3, which indicated that multifunctional nanocomposites could be constructed based on this strategy.

The PCDA molecule possesses a carboxylic group, which would stay at the outside of the resulting hydrophilic nanoparticles, thus allowing for further introduction of other functionalities. To evaluate such capability, we synthesized *p*-aminophenylboronic acid modified PCDA (PCDA-BA) by means of EDC/NHS coupling reaction (preparation procedures as well as the characterization was shown in Supplementary Figures 4–6). The obtained PCDA-BA would be further employed for surface modification of nanoparticles to achieve a selective targeting ability.

### Controllable Synthesis of Ultra-Bright ZAIS NPs

**Figure 3:**
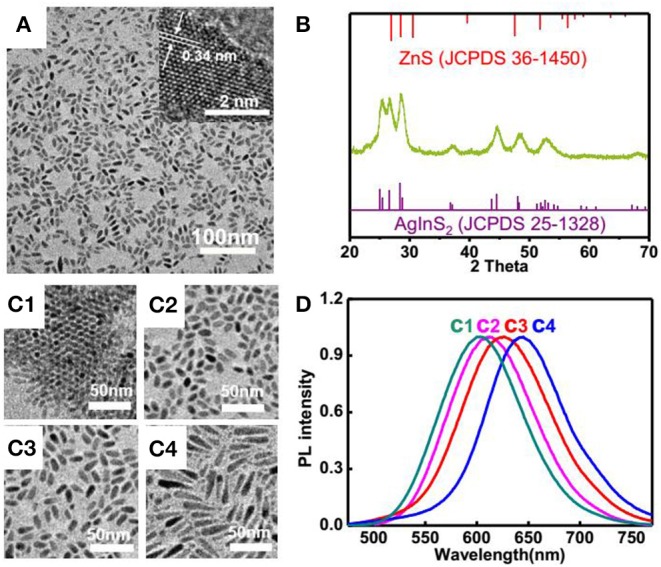
**(A)** TEM and HRTEM (inserted) images and **(B)** XRD patterns of ZAIS nanoparticles; **(C)** TEM images of ZAIS with different dosages of sulfur: **(C1)** 1.6 mg; **(C2)** 2.4 mg; **(C3)** 4.8 mg; **(C4)** 9.6 mg; **(D)** Normalized photoluminescence spectra of ZAIS with different dosages of sulfur.

In another aspect, we synthesized ZnS-AgInS_2_ nanocrystals, termed as ZAIS, through premixing In and Zn precursors with sulfur, followed by injection of silver precursors. The TEM image displayed a uniform rod-like structure and HRTEM presented a continuous lattice fringes throughout the particles with interplanar space of 0.34 nm (Song et al., 2016), suggesting a typical solid solution of ZAIS, which is consistent with observation of corresponding XRD patterns, shown in Figures 3A,B considering that the main peaks located in between that of ZnS and AgInS_2_ (Torimoto et al., 2007). It shall be noted that most of ZAIS reported in the literature have a spherical structure and for preparation of ZAIS with anisotropic shapes (Xie et al., 2009), it normally leaded to a mixture of particles with different shapes. Here, the afforded ZAIS display a uniform rod-like structure. In contrast to reported protocols that always directly mixed up all the corresponding metal salts at first hand, we premix indium and zinc salt with sulfur powder, followed by introduction of silver precursor. It is anticipated that the silver ion has strongest tendency for interaction with sulfur and adding it at later stage would afford more uniform nucleation, which then facilitate formation of uniform rod-like structure. Moreover, the morphologies could be readily modulated with the dosages of sulfur, as evidenced in Figure 3C and the emission peak wavelength showed a red-shift along the increase of sulfur dosage (Figure 3D). The resulting ZAIS nanocrystals displayed a high quantum yield, measured to be 78.9%, as shown in Supplementary Figure 7, with a long luminescence lifetime of 1.5 μs, which hold great potentials in terms of bioimaging.

### Stability and Cytotoxicity ZAIS@PCDA-BA NCs

**Figure 4:**
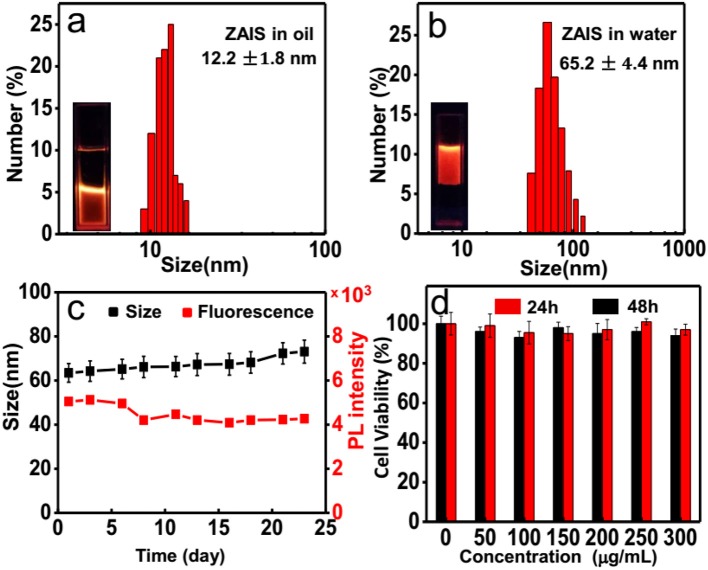
DLS results for the ZAIS@PCDA-BA NCs **(a)** before and **(b)** after surface modification. **(c)** Size distribution and photoluminescence intensity changes of ZAIS@PCDA-BA NCs along with time. **(d)** Cell viability tests of ZAIS@PCDA-BA NCs on HepG2 cell lines at different concentrations after incubation for 24 h (red) and 48 h (black), respectively.

Considering the excellent biocompatibility of ZAIS nanocrystals, here PCDA-BA were employed to transfer hydrophobic ZAIS into aqueous solution and meanwhile, introduced as targeting moieties. Following the as-reported protocols, ZAIS nanocrystals were mixed with PCDA and PCDA-BA under sonication treatment, followed by light irradiation. Similar to other nanoparticles, the TEM image of resulting nanocomposites (Supplementary Figure 8) indicated a monodispersed feature without aggregation and DLS results suggested a slight increase of hydrodynamic particle size after surface modification (Figures 4a,b). Though it has been reported polymerized PCDA backbone would induce a color change, here judging from the emission properties of ZAIS@PCDA-BA NCs, apparently it caused negligible effect on emission properties, shown in Supplementary Figure 9.

Moreover, the afforded hydrophilic ZAIS@PCDA-BA NCs are very stable as their fluorescence and size changes are well-maintained for over 3 weeks, as shown in Figure 4c. Prior to cell imaging, the cytotoxicity of ZAIS-BA NCs was tested by the MTT assay. As implied in Figure 4d, the results suggested that the cell viability maintained over 90% even after 48 h under the highest concentration (300 μg/mL), implying good biocompatibility of ZAIS@PCDA-BA NCs.

### Cell Imaging of ZAIS@PCDA-BA NCs

**Figure 5:**
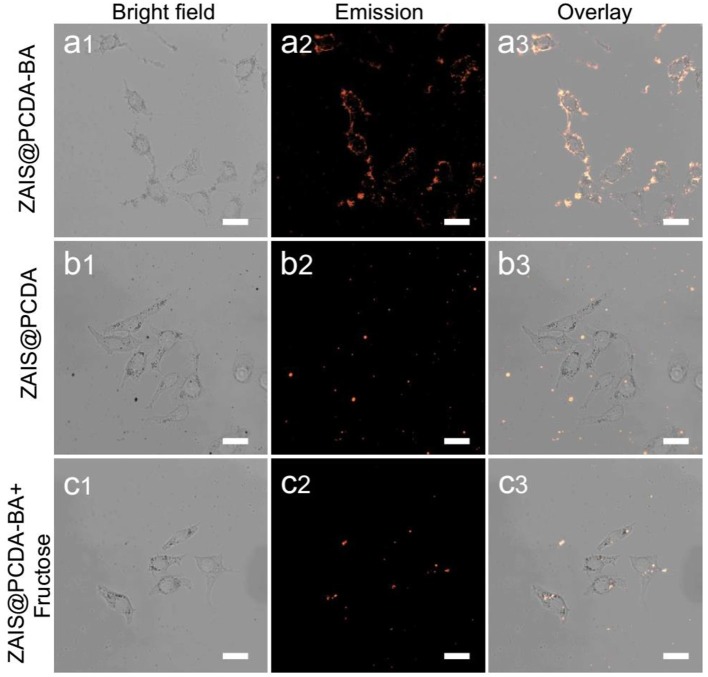
Confocal luminescence imaging of HepG2 cells incubated with **(a)** ZAIS@PCDA-BA NCs, **(b)** ZAIS@PCDA NCs, and **(c)** ZAIS@PCDA-BA NCs in the presence of D-fructose (0.1 M), respectively. Particles concentration: 300 μg/ml. Irradiation: 488 nm for ZAIS. Scale bar 25 μm.

Given that cancerous cells are known to overexpress sialic acid moieties on cell membrane surface, here the capacity of luminescent ZAIS@PCDA-BA NCs for selectively target cancer cells was explored. Initially, luminescent ZAIS@PCDA-BA NCs were incubated with cells for different time intervals and clearly it was found that as incubation time increased, more and more ZAIS@PCDA-BA NCs attached on the surface of cells, suggested by the gradually increased luminescent intensities, evidenced by Supplementary Figure 10. When incubation time increase to 12 h, endocytosis occurred (Supplementary Figure 11) since the whole cell were lighten up. Then, luminescent ZAIS with and without PCDA-BA modification were incubated with HepG2 cells. As shown in Figure 5a, the ZAIS nanocrystals that have PCDA-BA moieties on surface could specifically labeling on the membrane of HepG2 cells after incubation for 8 h. As for the control group in Figure 5b, it is hardly to observe luminescence, further demonstrating the selective targeting ability of PCDA-BA moieties toward cancerous cell membranes. In another aspect, ZAIS@PCDA-BA NCs were incubated with normal cell lines (HUVEC cells). Clearly, it is hardly to observe luminescence emission, as shown in Supplementary Figure 12, suggesting that ZAIS@PCDA-BA NCs displayed low affinity toward normal cells, which was reasonable since saccharide residues are only overexpressed in cancerous cells. In addition, it is known that boronic acid could bind with monosaccharide, which, we assumed, would have a competitive binding with sialic acid moieties on the cell surface. In this regard, we incubated ZAIS@PCDA-BA NCs with HepG2 cells in the presence of D-fructose, displayed as in Figure 5c, clearly the high concentration of D-fructose (0.1 M) would induce an off-targeting effect of ZAIS@PCDA-BA NCs toward cell membranes.

## Conclusions

In summary, we have developed a general and facile method for hydrophobic nanoparticles surface engineering with various shapes, sizes, and chemical compositions. By taking advantage of the *in-situ* polymerization of PCDA, we obtained surface functionalized nanocomposites with high stability in aqueous solution without obvious changing in proprieties such as luminescence, optical and magnetism. In addition, owing to the carboxylic group of PCDA, the functionalized nanocrystals are biocompatibility and bioconjugatable. As a proof-of-concept, PCDA-BA was used to functionalize ZAIS nanocrystals, which then was used for specific labeling cancerous cells. The method is simple and versatile, thus can be used as a general method for surface engineering of hydrophobic inorganic nanocrystals.

## Data Availability Statement

The raw data supporting the conclusions of this manuscript will be made available by the authors, without undue reservation, to any qualified researcher.

## Author Contributions

SG performed all the experiments. CG helped with the cell imaging experiments. HW and GT helped with the nanoparticle synthesis. SX drafted the manuscript. SX and LW supervised the project and were in charge of overall direction of the project. All authors contributed to the final version of the manuscript. All the authors have agreed with the manuscript for submission.

### Conflict of Interest

The authors declare that the research was conducted in the absence of any commercial or financial relationships that could be construed as a potential conflict of interest.
