# Supplementary material for: A Versatile Strategy for Surface Functionalization of Hydrophobic Nanoparticle by Boronic Acid Modified Polymerizable Diacetylene Derivatives
Source: Front Chem. 2019 Nov 1;7:734. doi: 10.3389/fchem.2019.00734 (PMC6839036; doi:10.3389/fchem.2019.00734)
Supplement: Supplementary file 1 [file Table_1.docx]

Supplementary Material

A Versatile Strategy for Surface Functionalization of Hydrophobic Nanoparticle by Boronic Acid Modified Polymerizable Diacetylene Derivatives

## Supplementary Figures

**Supplementary Figure 1.** The synthetic scheme for PCDA-BA (Compound **4**).


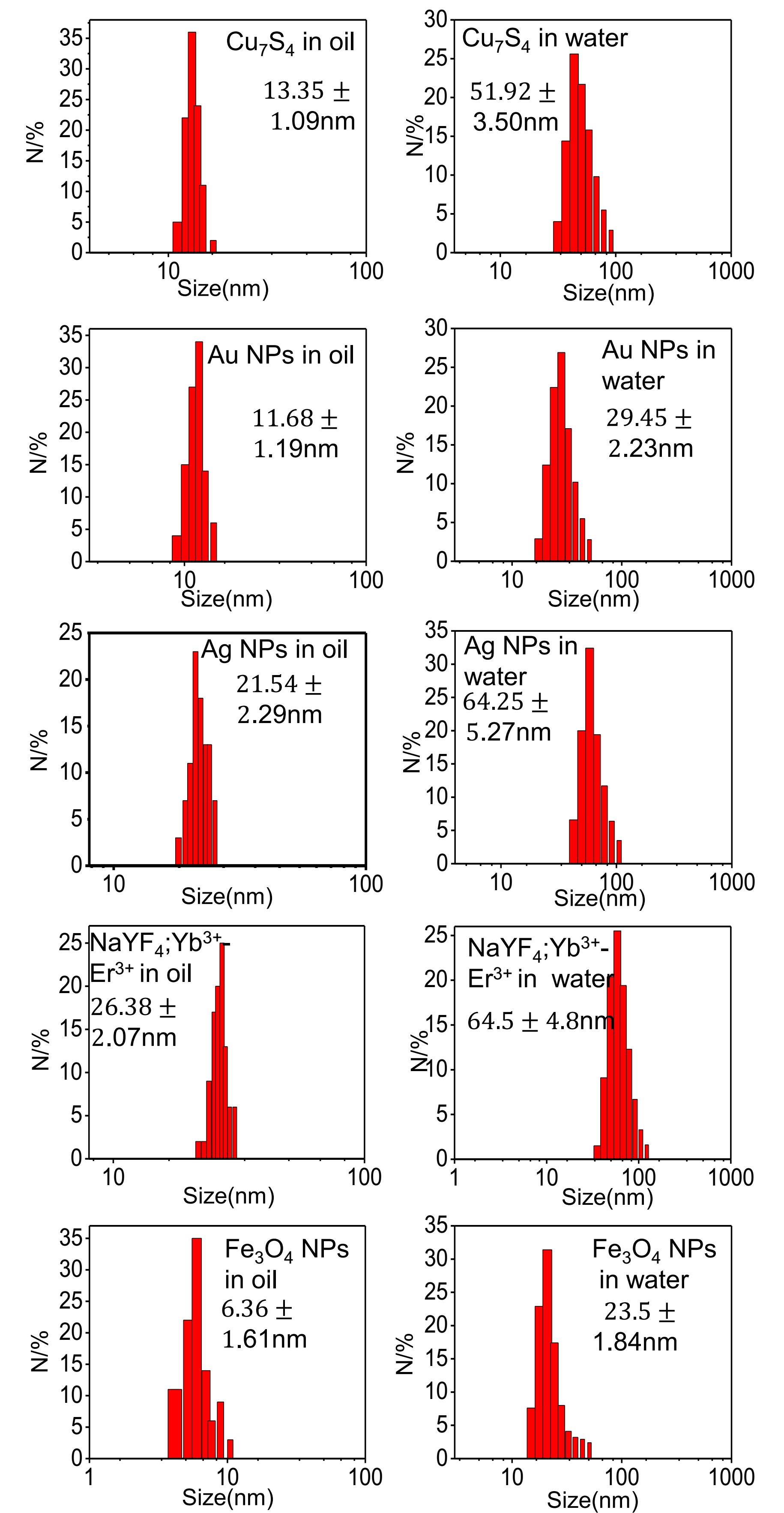


**Supplementary Figure 2.** Size distribution profiles obtained by dynamic light scattering (DLS) of nanoparticles before (left column) and after (right column) surface engineering (each nanocomposites containing single nanoparticles).


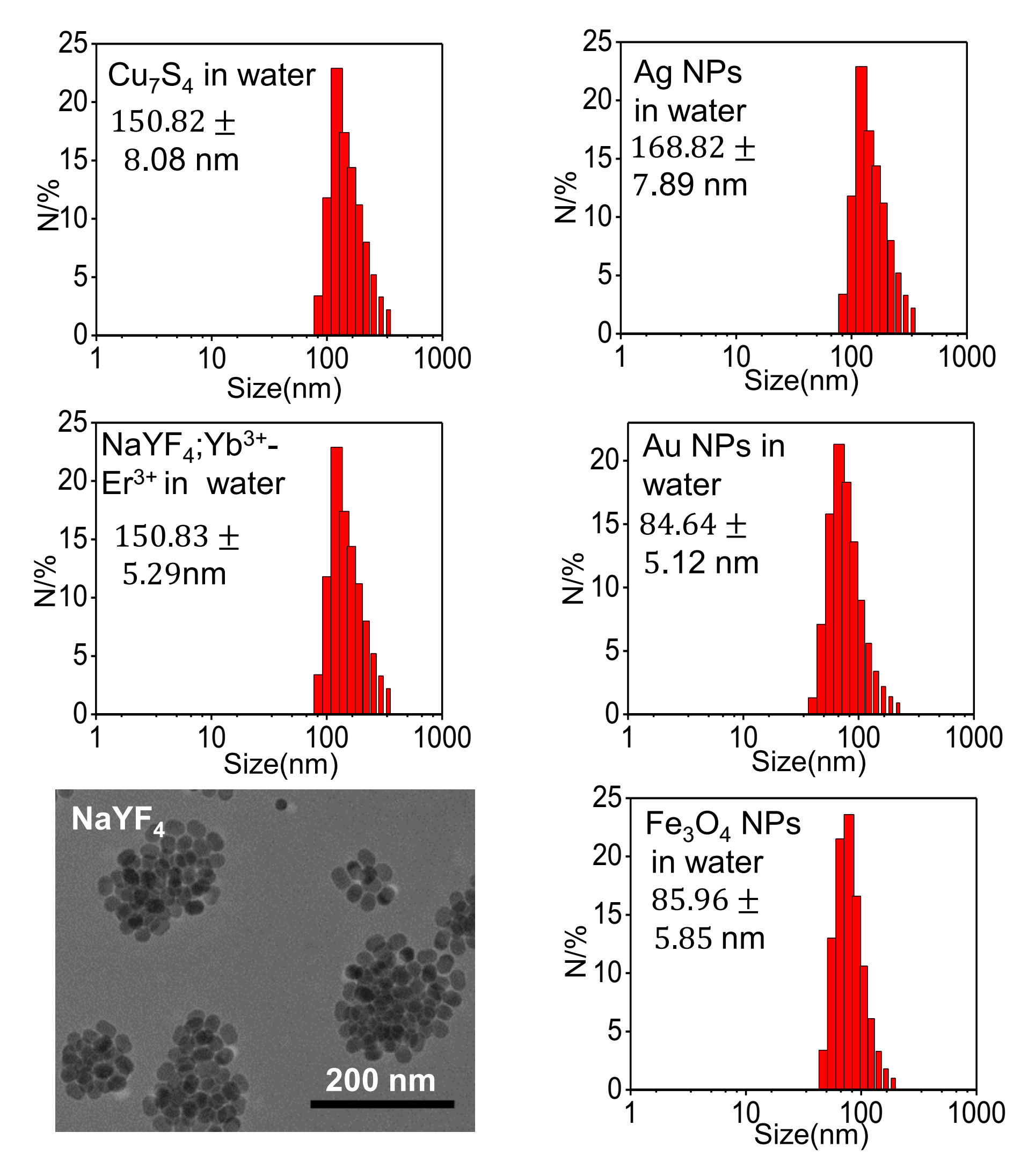


**Supplementary Figure 3.** DLS measurement results of nanocomposites containing multiple nanoparticles. The TEM image is typically for nanocomposites composed of multiple NaYF_4_ nanoparticles.


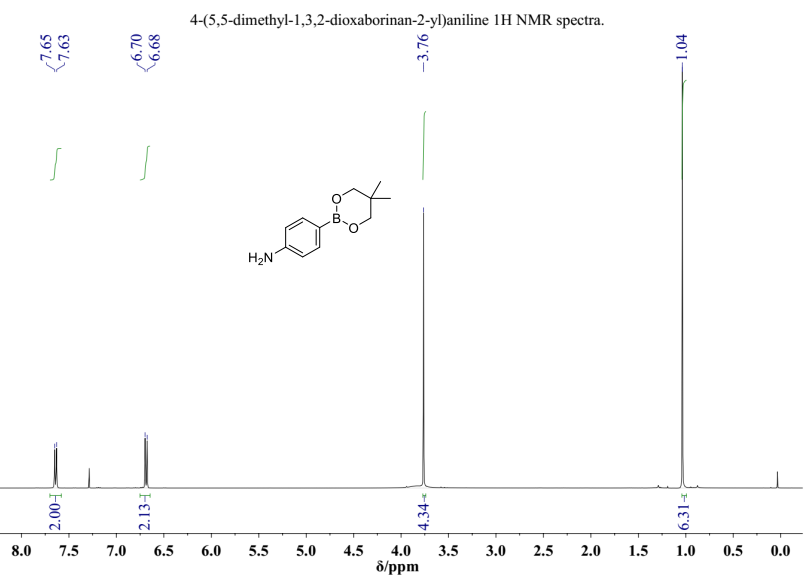


**Supplementary Figure 4.** ^1^H NMR spectrum of compound **2**.


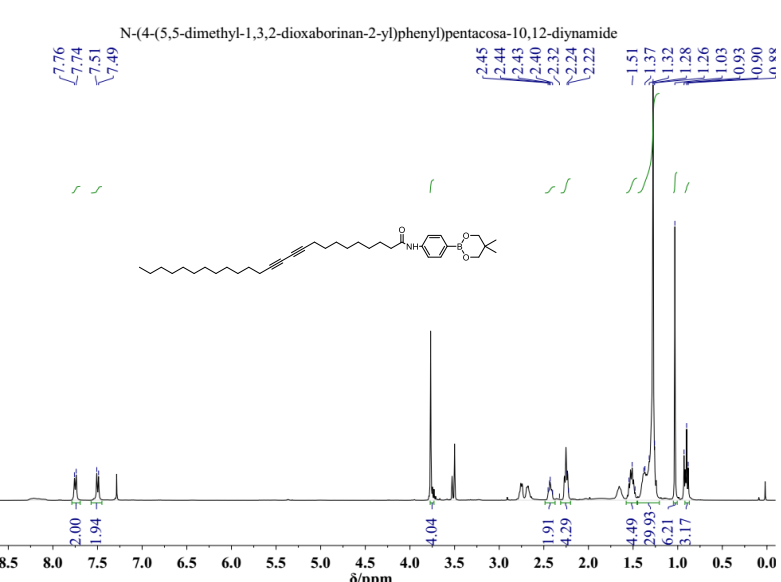


**Supplementary Figure 5.** ^1^H NMR spectrum of compound **3**.


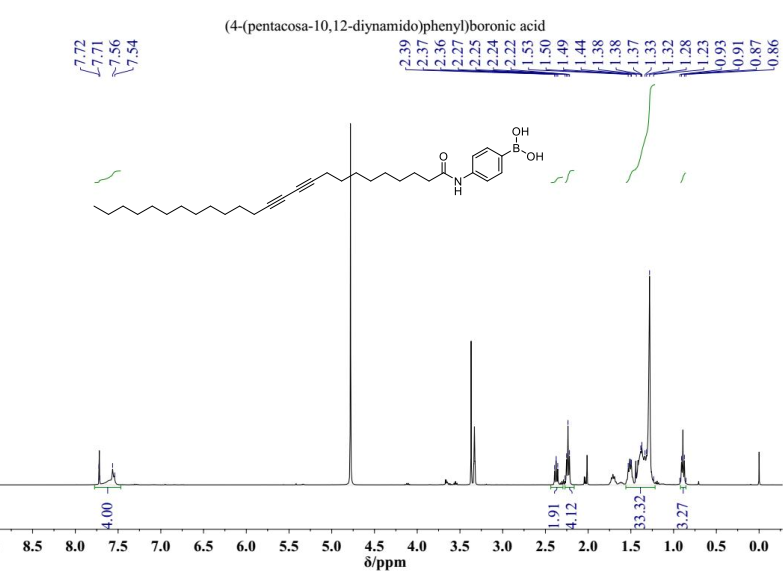


**Supplementary Figure 6.** ^1^H NMR spectrum of compound **4**.

**
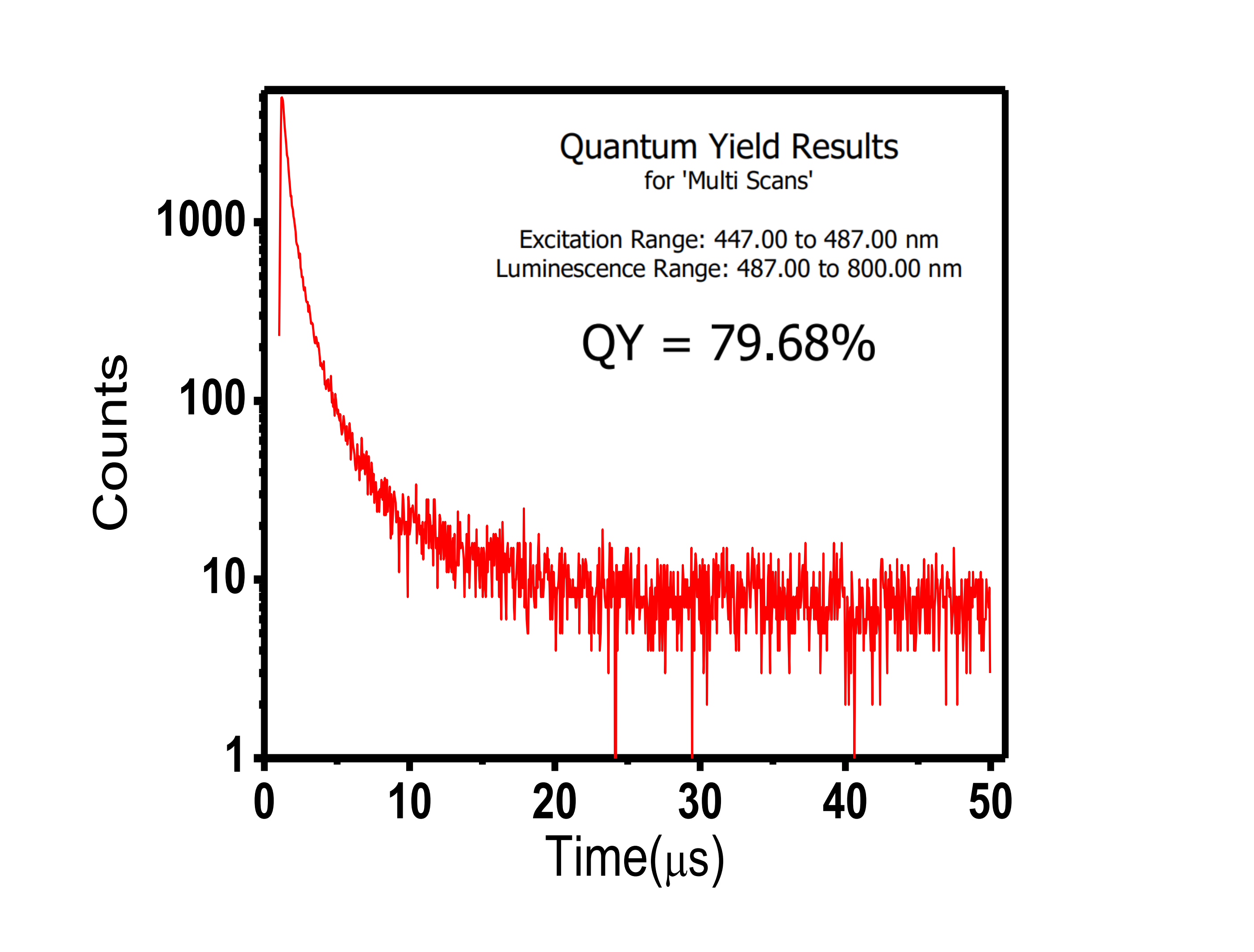
**

**Supplementary Figure 7.** Quantum yield and time resolved spectroscopy of ZAIS NPs.

**
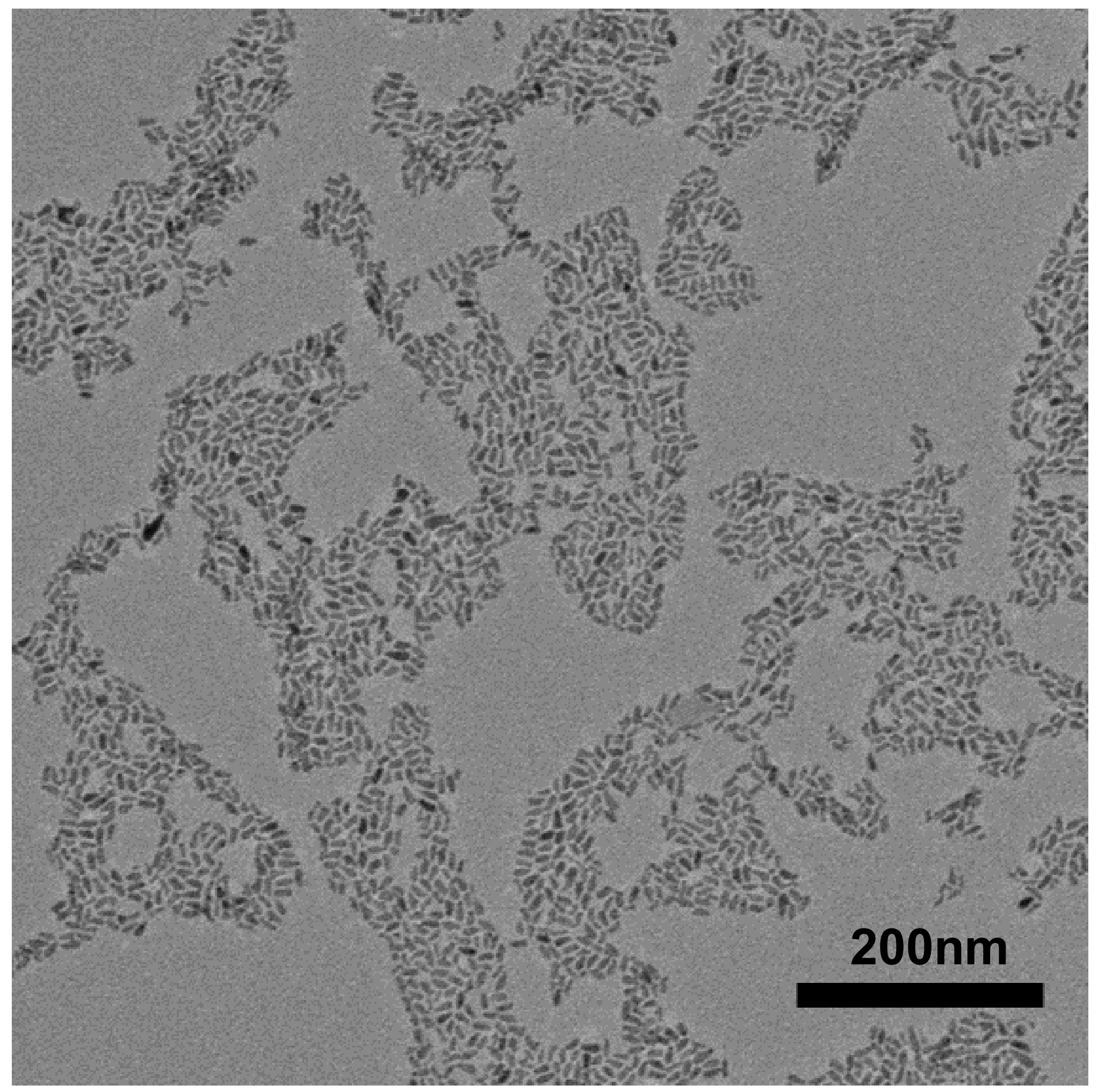
**

**Supplementary Figure 8.** TEM image for hydrophilic ZAIS@PCDA-BA NCs

**
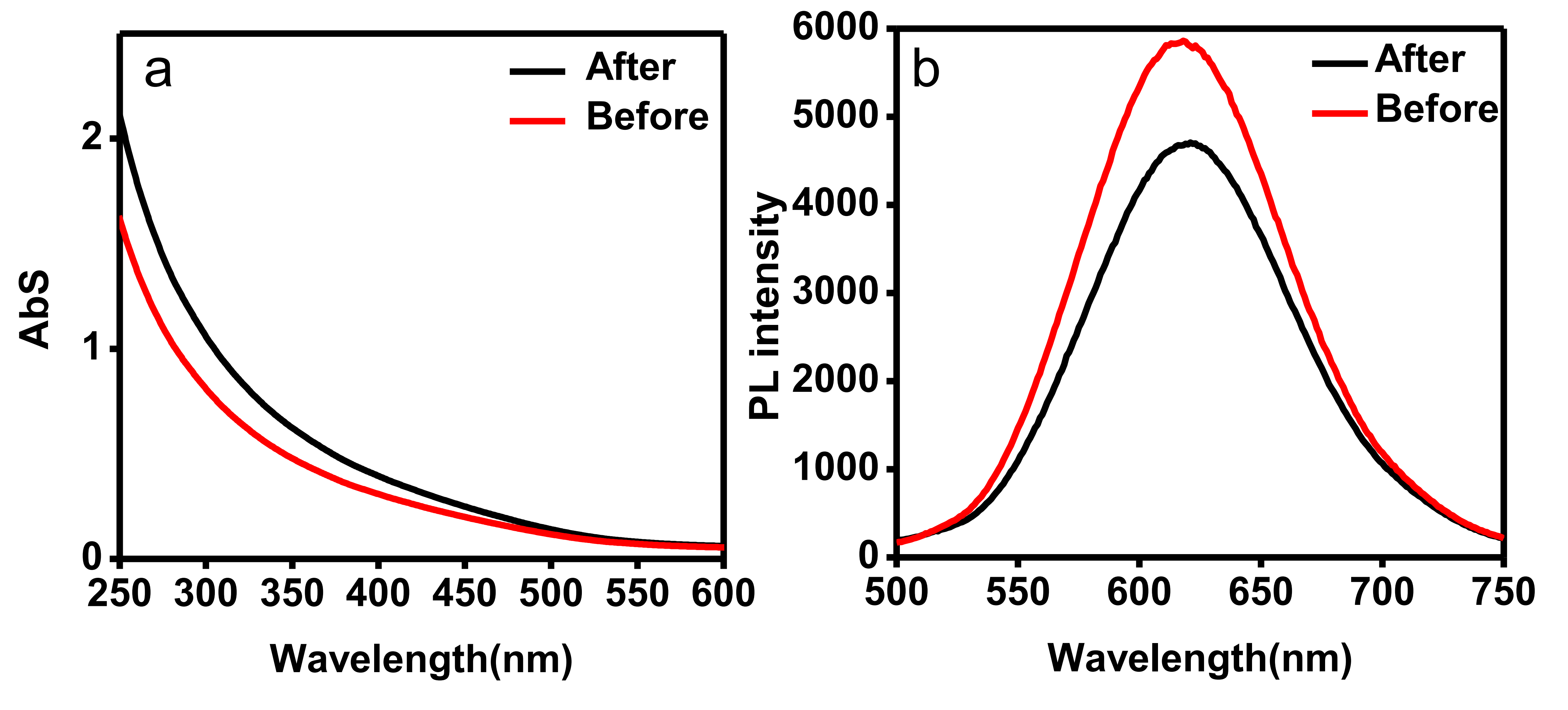
**

**Supplementary Figure 9.** Absorption spectra (a) and PL spectra (b) of ZAIS@PCDA-BA NCs before (red) and after (black) PCDA polymerization. Excitation wavelength at 440 nm.

**
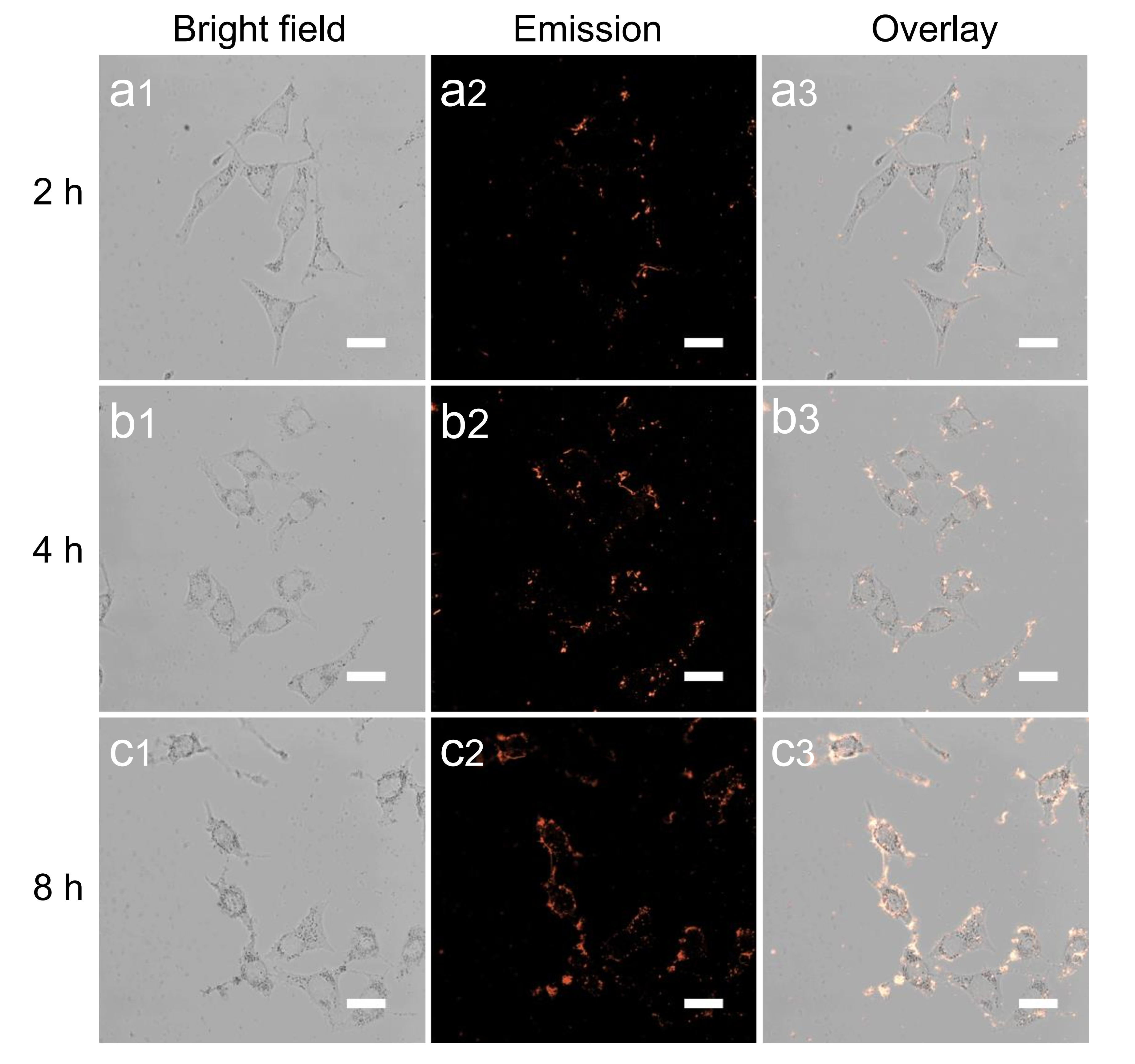
**

**Supplementary Figure 10**. Confocal luminescence images of HepG2 cells incubated with ZAIS@PCDA-BA NCs (a) 2 h, (b) 4 h and (c) 8 h, respectively. Particles concentration: 300 μg/ml. Irradiation: 488 nm for ZAIS. Scale bar 25 μm.


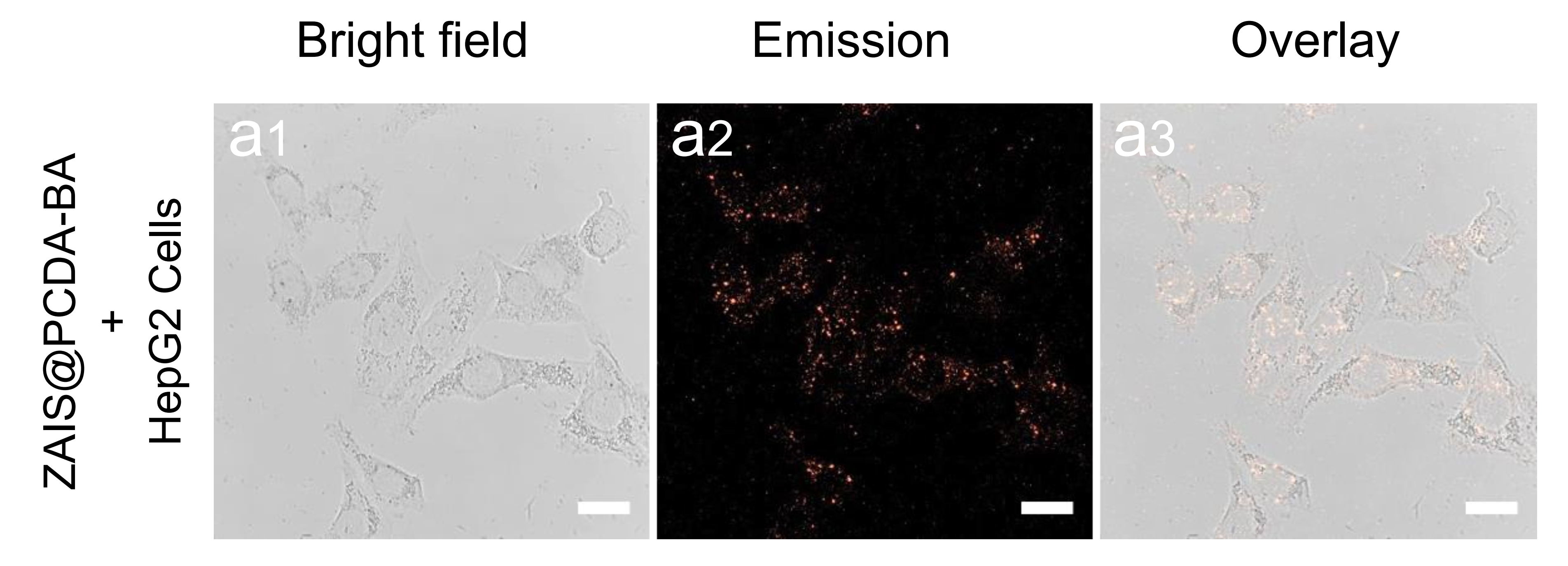


**Supplementary Figure 11**. Confocal luminescence imaging of HepG2 cells incubated with ZAIS@PCDA-BA NPs for 12h. Particles concentration: 300 μg/mL. Irradiation: 488nm for ZAIS. Scale bar 25 μm.


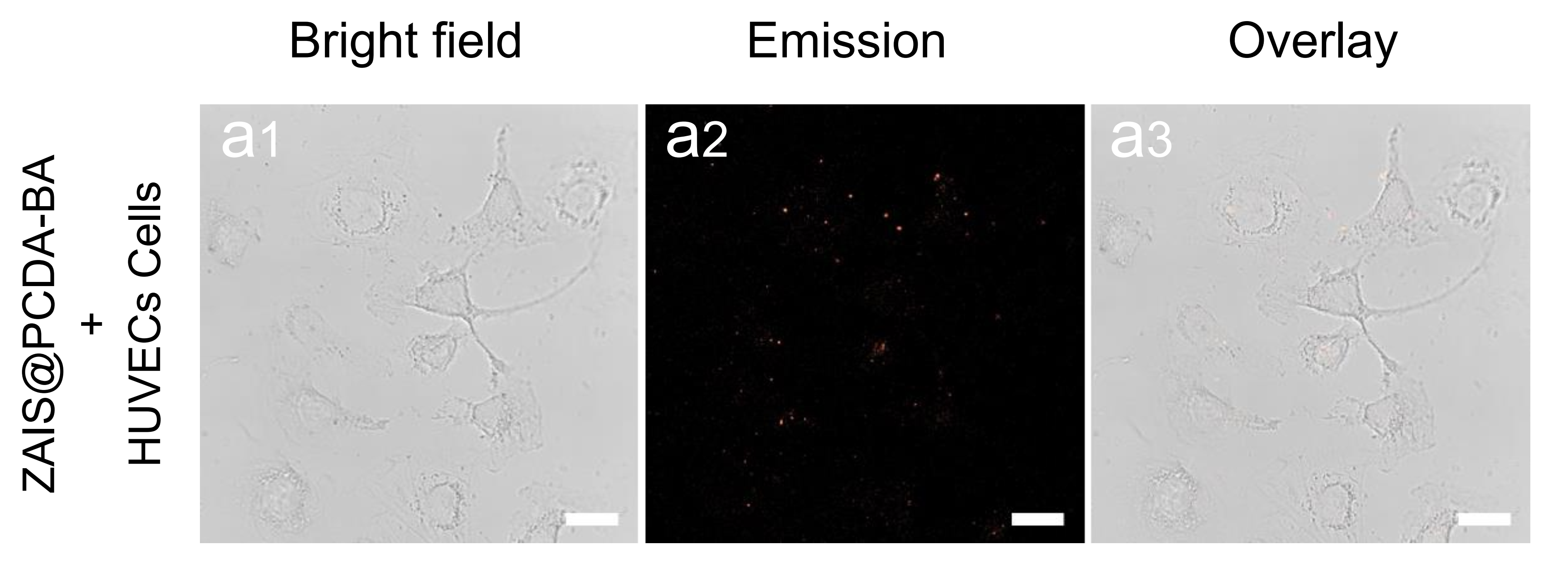


**Supplementary Figure 12** Confocal luminescence imaging of HUVECs Cells incubated with ZAIS@PCDA-BA NPs for 8 h. Particles concentration: 300 μg/mL. Irradiation: 488nm for ZAIS. Scale bar 25 μm.
